# Magnetic actuation and feedback cooling of a cavity optomechanical torque sensor

**DOI:** 10.1038/s41467-017-01380-z

**Published:** 2017-11-07

**Authors:** P. H. Kim, B. D. Hauer, T. J. Clark, F. Fani Sani, M. R. Freeman, J. P. Davis

**Affiliations:** Department of Physics, University of Alberta, Edmonton, AB Canada T6G 2E9

## Abstract

Cavity optomechanics has demonstrated remarkable capabilities, such as measurement and control of mechanical motion at the quantum level. Yet many compelling applications of optomechanics—such as microwave-to-telecom wavelength conversion, quantum memories, materials studies, and sensing applications—require hybrid devices, where the optomechanical system is coupled to a separate, typically condensed matter, system. Here, we demonstrate such a hybrid optomechanical system, in which a mesoscopic ferromagnetic needle is integrated with an optomechanical torsional resonator. Using this system we quantitatively extract the magnetization of the needle, not known a priori, demonstrating the potential of this system for studies of nanomagnetism. Furthermore, we show that we can magnetically dampen its torsional mode from room-temperature to 11.6 K—improving its mechanical response time without sacrificing torque sensitivity. Future extensions will enable studies of high-frequency spin dynamics and broadband wavelength conversion via torque mixing.

## Abstract (web summary)

Although optomechanics enables precision metrology, measurements beyond mechanical properties often require hybrid devices. Here, Kim et al. demonstrate that a ferromagnetic needle integrated with a torsional resonator can determine the magnetic properties and amplify or cool the resonator motion.

## Introduction

While the field of cavity optomechanics has enabled remarkable advances in measuring and manipulating nanomechanical resonators at, or approaching, their quantum limits^1,2^, the most attractive applications of optomechanics require integration with one or more additional systems^3^. For example, the first definitive test of quantum behaviour in a mechanical resonator was enabled by integration with a superconducting qubit^4^. Further experiments have demonstrated coupling between optomechanical resonators and atomic gases^5^, superfluids^6^, and magnetic materials^7,8^, with the possibility of coupling to small numbers of spins^9–12^ and even biological samples^13^. Such hybrid systems are potentially the key to microwave-to-telecom wavelength conversion at the quantum level^14^, and enable ultra-sensitive measurements of nanoscale and mesoscale samples^15^.

Unfortunately, most optomechanical systems are not amenable to integration with external samples. For example, two of the most impressive architectures for cavity optomechanics—the optomechanical crystal^2^ and the superconducting membrane^1^—are particularly difficult to directly integrate with condensed matter systems. In an optomechanical crystal, the optical mode significantly overlaps with the mechanical motion^2^—providing good optomechanical coupling but difficulty in placing a secondary system onto the mechanics without adversely affecting the optical mode. Likewise, the membrane resonators used with superconducting microwave cavities have extraordinarily small capacitor gaps and are highly tensioned^1^, neither of which are compatible with integration of an additional condensed matter system onto the mechanical membrane. There remains a need for carefully designed structures to allow for useful hybrid integration, such as the open geometry of 3D microwave cavities^16^, fabrication out of the condensed matter system under study^9,11,12^, or—as we use here—a modular structure of separate optical and mechanical components with tailorable coupling.

Here, we demonstrate integration of a mesoscopic ferromagnetic needle with a cavity optomechanical resonator, and show that this hybrid system can be used for magnetic field sensing, with a thermal limit of 0.12 A m^−1^ (150 nT); quantitative determination of the magnetization of the needle; and magnetic feedback cooling of the mechanical mode from room temperature to below 12 K—representing the first demonstration of feedback cooling with a magnetic cavity optomechanical system. The latter has significant potential applications as it allows for faster measurements of the mechanical resonator, but without sacrificing its sensitivity—as we demonstrate here explicitly. Such magnetic-feedback cooling will be useful for high-frequency measurements of collective spin excitations in nanoscale materials^15^. In addition, through the mixing that occurs with torsional magnetic actuation^15^, this system could enable widely tunable, broadband, microwave-to-telecom wavelength conversion^17^.

## Results

### Hybrid device design

**Fig. 1:**
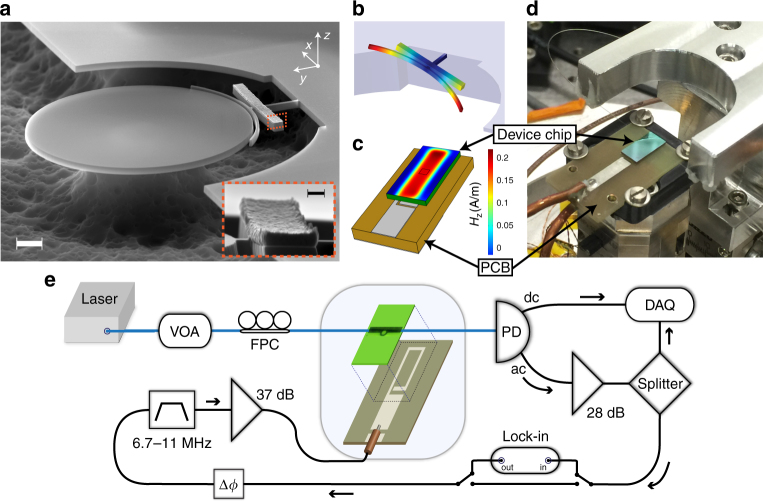
Magneto-optomechanical system. **a** Scanning electron microscope (SEM) image, tilted to 70°, showing the optomechanical torque sensor, with an 8.8 μm diameter optical WGM microdisk evanescently coupled to an arced torsional resonator. Scale bar is 1 μm. Inset shows the integrated trilayer needle of Cr, Fe, and Cr, which has a total thickness of 83 nm (4.39 μm long and 410 nm wide), near the end of the torsion arm. Scale bar is 100 nm. **b** A simulation of the torsional mode at *Ω*_m_/2*π* = 7.2 MHz (*I*_eff_ = 4.4 pg μm^2^, *m*_eff_ = 445 fg), which can be driven by a magnetically induced torque along the *y*-axis. **c** Simulation of the out-of-plane magnetic field along the *z*-axis at the top surface of the device chip, with 1 mA of applied current. The device position is indicated by the black square, chosen for large field strength and relative field uniformity. **d** Photograph and **e** schematic of the optomechanical transduction and magnetic feedback. The phase, *ϕ*, of the feedback signal is measured using a high-frequency lock-in amplifier and is varied by the length of coaxial cable. The shaded blue region depicts the contents inside the vacuum chamber (see Methods): the optomechanical chip, the PCB drive chip, and the dimpled tapered fibre. VOA variable optical attenuator, PD photodetector, DAQ data acquisition system, FPC fibre polarization controller

One of the main considerations in the design of hybrid systems is taking advantage of the best properties of the individual systems, without sacrificing their performance during integration. Hence, we have chosen to directly integrate a magnetic structure onto our mechanical resonator. The architecture of our magneto-optomechanical system is shown in Fig. 1. The mechanical structure, with a low effective moment of inertia torsional mode (see Fig. 1b), is separated from the evanescent field of a whispering-gallery-mode (WGM) optical cavity by an 87 nm vacuum gap. A platform for the magnetic sample was designed near the end of the torsion arm, amplifying the magnetic actuation of the torsional resonator^18^, yet is sufficiently far from the evanescent field of the WGM such that its optical properties are unaffected (*Q*_opt_ = 5.3 × 10^4^). On this platform we have deposited a ferromagnetic sample, which enables the mechanical motion to be driven, amplified, or dampened, by an alternating (ac) external magnetic field. Details on device fabrication and measurement can be found in the Methods section.

### Magnetic actuation

To test the responsiveness of the torsional resonator to magnetic actuation, we first characterized the driven response. Since the ferromagnetic needle is magnetized, applying an external magnetic field, **H**, perpendicular to its magnetic moment, **m**, causes a torque:

1 $$\tau = {\bf m} \times \mu _0{\bf H}.$$

**Fig. 2:**
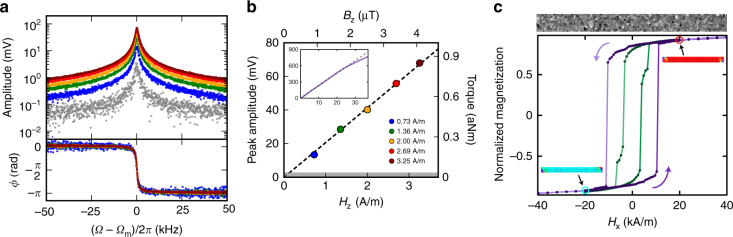
Magnetic actuation and sensing. **a** Amplitude of the thermomechanical (grey) and magnetically actuated (coloured) torsional resonance, with corresponding phase of the driven traces. Colours in **a** correspond to the drive magnetic field plotted in **b**, which shows the relationship between the peak mechanical signal amplitude (left axis) and calibrated torque (right axis), vs. the magnetic drive. The grey band represents the thermomechanically limited minimum field sensitivity of 0.12 A m^−1^, corresponding to a thermal torque of 32 zNm. Inset, with the same axes, shows the deviation of the continuously measured peak response (purple) from the linear fit extrapolated from low field (dashed). Above 25 A m^−1^ the optical resonance is shifted due to heating from the drive chip. **c** Simulated magnetization (normalized to *M*_s_) hysteresis (see Methods for details) with in-plane domain structure at circled points. At high fields the needle is nearly saturated, with triangular domains at each end. As the field is lowered these domains move toward the centre, reducing the net magnetization. For a uniform iron film (green trace) at zero field, the remanent magnetization corresponds to 79% of the saturation moment. Adding polycrystalline grains, as shown above **c**, increases the remanent magnetization to 85%, as seen in the purple trace

Here, the magnetic moment is along the *x*-axis. We apply an orthogonal ac magnetic field along the *z*-axis that generates a torque along the *y*-axis, i.e., the torsional axis of the resonator, Fig. 1. Sweeping the frequency of the magnetic drive through the mechanical resonance results in magnetically actuated torsional motion, as shown in Fig. 2. The increase in peak amplitude of the mechanical spectrum is linear with magnetic drive until 25 A m^−1^ (31 μT), shown inset to Fig. 2b. This allows us to calibrate the peak amplitude in terms of the external magnetic field, and determine the thermomechanically limited minimum field sensitivity of 0.12 A m^−1^ (150 nT). Therefore as a magnetic field sensor, this system is linear over two orders of magnitude, from 0.12 A m^−1^ to 25 A m^−1^, with a responsivity of 168 ± 2 μrad (A m^−1^)^−1^. Note that lowering the temperature of the resonator would improve its dynamic range and sensitivity^18^. As can be seen in Eq. (2) below, such an improvement stems from both the lowering thermal torque noise and the intrinsic damping rate at low temperatures, although the exact mechanism for decreased mechanical damping is complex^19^.

### Torque magnetometry

Using the thermomechanical calibration method outlined in ref. ^20^, we determine the measured Brownian torsional motion of the mechanical resonator, and hence the thermal torque applied to it. The right axis of Fig. 2b shows the calibrated torque measured at the mechanical resonance frequency, with a minimum (thermal) torque of 32 zNm. In addition, the straight line fit to the data in Fig. 2b using Eq. (1) allows extraction of the total magnetic moment of the ferromagnetic needle, |**m**| = (2.06 ± 0.02) × 10^−13^ A m^2^, or equivalently, (2.22 ± 0.02) × 10^10^ *μ*_B_. Taking into account the measured volume of the iron portion of the needle, *V* = (1.21 ± 0.09) × 10^−19^ m^3^, we find a magnetization, *M*, of 1710 ± 140 kA m^−1^. This compares favourably with the room temperature saturation magnetization of bulk iron, $M_{\mathrm{s}}^{{\mathrm{iron}}} = 1710$ kA m^−1^ ^21^. Furthermore, the large measured magnetization suggests that domain wall pinning—resulting from polycrystalline grains—plays an important role, as it serves to increase the remanent magnetization above that of a uniform film (Fig. 2c and Methods).

### Feedback cooling

Beyond using an ac current source for magnetic actuation, we can also use the measured mechanical signal to amplify or dampen the resonator motion. This type of feedback has been used in the cantilever sensing community, where amplification allows cantilever motion to be detected in highly damped conditions such as liquid environments^22^ and cooling allows for faster measurements to be performed without compromising sensitivity, due to the lower mechanical quality factor of the resonator, *Q*_m_. It is important to note that feedback cooling (or amplification) cannot increase the torsional sensitivity of the resonator, as the thermally limited torque sensitivity on resonance is given by

2 $$S_{\mathrm{\tau }} = 4k_{\mathrm{B}}T\Gamma I_{{\mathrm{eff}}},$$

where *T* is the mechanical mode temperature and *Γ* = *Ω*_m_/*Q*_m_ is the mechanical damping rate^18^. Feedback cooling lowers *T* at the same rate that it increases *Γ*, therefore the torque sensitivity is unchanged. Nonetheless, the change in the effective *Q* can make a substantial difference in the visibility of the mechanical signal and the ring-up time of the mechanical mode. It is the decrease in the ring-up time that we find particularly appealing for torque magnetometry of condensed matter systems. For example, if one is interested in collective spin ensembles, in nano-magnetic^15^ or mesoscale superconducting^18^ test samples, a lower *Q* resonator—while maintaining an equivalent torque sensitivity—allows for measurement of higher-frequency dynamics of spin modes^15^.

To test the performance of feedback amplification and cooling in our magneto-optomechanical system, we implement the scheme shown in Fig. 1e. Half of the ac signal, measuring the thermomechanical motion, is sent to a high-frequency data acquisition system, while the other half is phase shifted, bandpass filtered around the mechanical resonance of interest, and amplified, before being sent to the *z*-axis drive coil. The phase shift is particularly important, as can be seen from the equation of motion for a damped, driven harmonic oscillator subject to a feedback force:

3 $$\ddot z + \Gamma _{\mathrm{i}}\dot z + {\mathrm{\Omega }}_{\mathrm{m}}^2z = \frac{{F_{{\mathrm{th}}}}}{{m_{{\mathrm{eff}}}}} - g_{{\mathrm{fb}}}\Gamma _{\mathrm{i}}{\rm e}^{i\left( {\phi + \pi /2} \right)}{\kern 1pt} \left( {\dot z + \dot z_{\mathrm{n}}} \right),$$

where *Γ*_i_ is the intrinsic mechanical dissipation rate. The first term on the right hand side is proportional to the thermal force, *F*_th_, acting on the mechanical resonator, while the second is related to the applied feedback force. Here, *g*_fb_ is the gain of the feedback loop, *ϕ* is the measured phase difference between the drive and the displacement, and *z*_n_ is the measurement noise^23,24^. The resulting total mechanical dissipation can therefore be written as:

4 $$\Gamma _{{\mathrm{tot}}} = \Gamma _{\mathrm{i}}(1 + g_{{\mathrm{fb}}}{\kern 1pt} {\mathrm{cos}}(\phi + \pi {\mathrm{/}}2)).$$

**Fig. 3:**
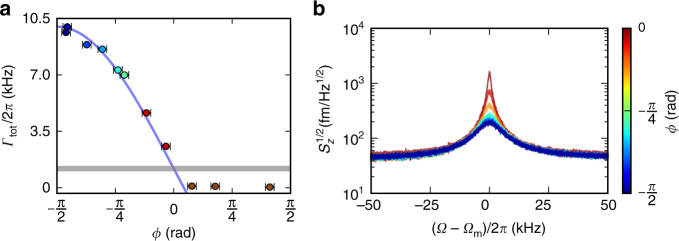
Magnetic feedback cooling and self-oscillations. **a** The total dissipation, *Γ*_tot_/2*π*, as a function of phase in the feedback loop, shows excellent agreement with a fit to Eq. (4) (blue curve). The optical power is 4.1 μW at the device. Error bars represent the standard deviation in the phase noise^37^, as measured by the Zurich lock-in amplifier. Maximum damping occurs at −*π*/2, and self-oscillation^25^ occurs for measured phases greater than zero (brown). The grey horizontal line shows the intrinsic dissipation, *Γ*_i_/2*π* = 930 Hz, identical to the total dissipation at *ϕ* = 0. **b** The same colour-coded data represented in terms of calibrated displacement spectral densities, for *ϕ* < 0

The phase controls whether the feedback gain results in cooling (*ϕ* = −*π*/2), amplification (*ϕ* = *π*/2), or does not modify the damping rate (*ϕ* = 0). In Fig. 3, we show how the measured dissipation, *Γ*_tot_, is affected by the phase in the feedback loop. These measurements were performed with a moderate optical power of 4.1 μW at the microdisk, resulting in a feedback gain of *g*_fb_ = 7.4 ± 0.1, extracted from fitting Eq. (4) to the *ϕ* < 0 data in Fig. 3a. We note that the gain was kept moderate in these measurements to prevent overloading of the electronic components in the region of feedback amplification, *ϕ* > 0 data in Fig. 3a, where the mechanical linewidth narrows and results in induced self-oscillation^25^.

**Fig. 4:**
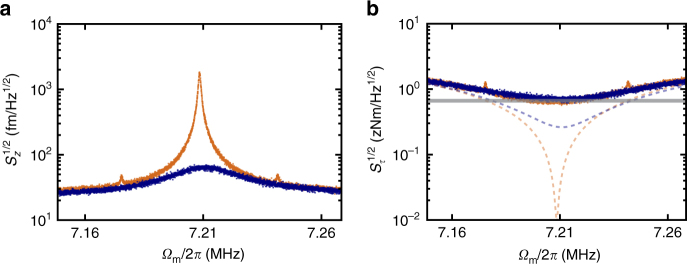
Optimal feedback cooling and torque sensitivity. **a** Increasing the power in the optical cavity enables sensitive mechanical transduction, lowering the imprecision noise floor to 25 fm Hz^−1/2^ (orange trace). The theoretical minimum temperature achievable with this noise floor is 8.6 K, and with a feedback phase of −*π*/2 and a feedback gain of 29 we find we are able to cool to 11.6 K (blue trace). **b** The calibrated torque sensitivity before (orange) and after (blue) feedback damping to a *Q*_m_ of just 260. Dashed traces correspond to torque sensitivities in the absence of thermal noise, which is given by the grey line. The device maintains a minimum torque sensitivity of 0.58 zNm Hz^−1/2^, regardless of whether or not feedback is applied. This demonstrates the potential advantage of torque magnetometry with this system, which can maintain state-of-the-art torque sensitivity with short mechanical ring-up times

To demonstrate our full feedback cooling efficacy, we increase the optical power at the microdisk to 18 μW, which results in a shot-noise-limited noise floor of $\sqrt {S_{\mathrm{z}}^{{\mathrm{imp}}}}$ = 25 fm Hz^−1/2^. This optical power produces our largest feedback gain, as shown in Fig. 4, increasing the total dissipation from *Γ*_i_/2*π* = 930 Hz (*Q*_m_ = 7760) to *Γ*_tot_/2*π* = 28,000 Hz (*Q*_m_ = 260). Evaluating Eq. (4) at *ϕ* = −*π*/2, a quantitative value for the feedback gain can be determined as *g*_fb_ = *Γ*_tot_/*Γ*_i_ − 1 = 29. Furthermore, we can infer the reduced mechanical mode temperature using the relation:^23,26^

5 $$T_{{\mathrm{fb}}} = \left( {T + \frac{{m_{{\mathrm{eff}}}{\mathrm{\Omega }}_{\mathrm{m}}^2\Gamma _{\mathrm{i}}g_{{\mathrm{fb}}}^2S_{\mathrm{z}}^{{\mathrm{imp}}}}}{{4k_{\mathrm{B}}}}} \right)\frac{1}{{1 + g_{{\mathrm{fb}}}}},$$

for which we find we can cool from room temperature to a value of *T*_fb_ = 11.6 ± 0.1 K. Note that the second term in Eq. (5) arises due to the fact that feeding back imprecision noise onto the resonator will act to heat the mechanical mode. The reduced mode temperature calculated from Eq. (5) is in reasonable agreement with that determined from the relative areas under the power spectral densities, which gives a mode temperature of 13.17 ± 0.02 K.

## Discussion

Comparing with the theoretical minimum achievable temperature using feedback cooling, which for high temperatures ($k_{\mathrm{B}}T \gg \hbar {\mathrm{\Omega }}_{\mathrm{m}}$) and large gain ($g_{{\mathrm{fb}}} \gg 1$) is given by:

6 $$T_{{\mathrm{min}}} = \sqrt {\frac{{m_{{\mathrm{eff}}}{\mathrm{\Omega }}_{\mathrm{m}}^2\Gamma _{\mathrm{i}}TS_{\mathrm{z}}^{{\mathrm{imp}}}}}{{k_{\mathrm{B}}}}} ,$$

we find *T*_min_ = 8.6 K for the values reported here. Note that this minimum temperature would be reached at a feedback gain of

7 $$g_{{\mathrm{fb}},{\mathrm{opt}}} = \sqrt {\frac{{4k_{\mathrm{B}}T}}{{m_{{\mathrm{eff}}}{\mathrm{\Omega }}_{\mathrm{m}}^2\Gamma _{\mathrm{i}}S_{\mathrm{z}}^{{\mathrm{imp}}}}}} ,$$

providing the optimal balance between cooling the mechanical motion via the cold damping feedback force and heating by feeding noise back onto the mechanical resonator. We calculate *g*_fb,opt_ = 68 using the experimental parameters from the data in Fig. 4, approximately a factor of two above the measured value. The result is a reduced temperature 3 K above the fundamental limit for the experiment performed here.

It is worth noting that, as stated earlier, the torque sensitivity is not improved (or substantially degraded) by feedback cooling. We show in Fig. 4b the calibrated torque sensitivity using the room temperature data before cooling, and after cooling (damping) the torsional mode to 11.6 K. Despite dramatically different dissipation rates and mechanical mode temperatures, they have identical torque sensitivities, with a minimum value of 0.58 zNm Hz^−1/2^—a factor of two better than the only other room temperature cavity optomechanical torque sensor with an integrated nano-magnetic sample^8^. Note that, while cavity optomechanical resonators with integrated magnetostrictive materials, such as the one in ref. ^27^, have achieved significantly better magnetic field sensitivity, they are not torque sensors and have no comparable torque sensitivity.

In summary, we have successfully integrated a ferromagnetic sample with a cavity optomechanical torsional resonator. First, we used this magneto-optomechanical system as a magnetic field sensor with a linear response from 150 nT to 31 T, and a responsivity of 0.134 ± 0.003 rad mT^−1^. Next, we quantitatively determined the properties of the magnetic sample, showing the potential of this system for studying mesoscopic condensed matter samples. And finally, we showed that using magnetic actuation, the resonator motion could be driven into self-oscillation or cold-damped to below 12 K. Future experiments could extend measurements to high frequency using torque-mixing^15^, low temperatures for superconducting samples and enhanced sensitivity^18^, or explore quantum spin tunnelling^28^ and exotic magnetic excitations such as those in topological systems^29,30^.

## Methods

### Device fabrication

Starting with a silicon-on-insulator (SOI) chip, having a single-crystal silicon device layer of thickness 250 nm, fabrication requires two e-beam lithography (EBL) steps. First, EBL is used to pattern the silicon device shown in Fig. 1a using ZEP-520a positive resist on a 30 kV EBL system (RAITH-150TWO). This is followed by a reactive ion etch (C_4_F_8_ and SF_6_) to transfer the pattern to the silicon layer. Afterwards, a second EBL process, with careful alignment, is used to pattern a PMMA bilayer resist in order to define the area for subsequent metal deposition. Electron-beam bombardment was used to deposit an 83 nm thick trilayer of Cr, Fe, and Cr. The purpose of the first 8 nm thick Cr layer is for adhesion to the silicon, whereas the last Cr layer, of equal thickness, serves as a capping layer to protect the iron from oxidation. Deposition took place at a pressure of 1.2 × 10^−7^ Torr and a deposition rate of 0.9 Å s^−1^ at 19 °C to minimize oxidation. During deposition, a rare-earth magnet was placed directly underneath the 5 mm × 10 mm SOI chip to preferentially orient the iron magnetization direction along the length of the needle (*x*-axis in Fig. 1a). The magnetic field strength at the position of the device during deposition was measured to be 94 kA m^−1^.

After lift-off of the PMMA and deposited metal using N-methyl-2-pyrrolidone, the chip is placed in a vapour HF system (MEMSstar) to etch the sacrificial SiO_2_ layer. Use of the HF vapour etch, instead of a buffered oxide etch, simultaneously avoids both problems of stiction and etching of the iron sample. It is also worth mentioning that the metallic needle cannot be arbitrarily long, as the stress induced during deposition causes deformation of the silicon resonator, apparent in Fig. 1a.

### Optomechanical measurement

System characterization is performed using a tuned-to-slope optomechanical detection scheme^18,31^. That is, as the torsional resonator moves in the evanescent field surrounding the WGM microdisk, the wavelength of the optical resonances are shifted. This encodes information about the mechanical motion in the optical transmission through the cavity, measured using laser light from a dimpled tapered fibre^32,33^. The dimpled-tapered fibre has a radius of curvature of 70 m, and touches the optical microdisk for stability and to ensure over-coupling (*κ*_e_/*κ* = 0.8). The device presented here has an optomechanical coupling coefficient of *g*_0_ = (d*ω*_c_/d*z*)*z*_zpf_ = 38 kHz, where *ω*_c_ is the optical cavity resonance frequency and the mechanical zero-point fluctuations are given by $z_{{\mathrm{zpf}}} = \sqrt {\hbar {\mathrm{/}}2m_{{\mathrm{eff}}}{\mathrm{\Omega }}_{\mathrm{m}}} = 51{\kern 1pt} \,{\mathrm{fm}}$. The reasonably large *g*_0_ enables measurement of the mechanical motion down to an imprecision noise-floor of 25 fm Hz^−1/2^. During measurement, the device chip is mounted on a printed-circuit-board (PCB) with two axes of orthogonal magnetic drive coils^15^, Fig. 1, although in the present experiment we only apply magnetic excitation along the *z*-axis. We use a high-frequency lock-in amplifier to drive current through the *z*-axis excitation coil. Measurements are performed in a room-temperature optical-access vacuum chamber at 1 × 10^−5^ Torr^33^.

For the data presented in Fig. 3, the phase was varied by adding calibrated lengths of coaxial cable to the feedback loop, and was measured using the lock-in amplifier at a frequency just below the mechanical resonance frequency.

It is worth noting that, despite the reasonably large *g*_0_ of our device, optomechanical damping is insignificant compared to feedback damping. For example, at the highest optical powers used here, feedback cooling results in an increased dissipation of *Γ*_fb_/2*π* = (*Γ*_tot_ − *Γ*_i_)/2*π* ≈ 27,000 Hz, whereas the maximum optomechanical damping that is possible is just *Γ*_om_/2*π* = 29 Hz for our device parameters^34^.

### Magnetic drive calibration

A Tektronix CT-2 current probe was used to calibrate the current output of the Zurich lock-in amplifier, which is then converted into a magnetic field (**H**_z_) at the position of the torsional device with the aid of a finite element simulation of the magnetic field, shown in Fig. 1c.

### Magnetic simulation

A GPU-accelerated open-source programme, mumax (version 3.9), was used to simulate the zero-temperature properties of a ferromagnetic needle with the as-fabricated dimensions of 4390 × 410 × 67 nm^3^ and the bulk properties of iron: exchange stiffness constant *A*_ex_ = 13.3 pJ m^−1^ and the *T* = 0 saturation magnetization *M*_s_ = 1740 kA m^−1^ ^21^, with a cell size of 10 × 10 × 6.7 nm^3^. While the simulation does not take into account the effects of temperature, hence the *T* = 0 value of the saturation magnetization is used, at room temperature the saturation magnetization is expected to be reduced by 2% to 1710 kA m^−1^ due to spin waves^35^. Magnetocrystalline anisotropy is neglected on account of the large aspect ratio, which makes shape anisotropy dominant^36^.

The hysteresis loop starts at a large positive field, 80 kA m^−1^, and the field is swept to −80 kA m^−1^, finally returning to the original positive field. A portion of the hysteresis loop, from −40 to 40 kA m^−1^ is presented in Fig. 2c. Hysteresis loops were simulated for both a uniform film, and one with a polycrystalline grain size of 40 nm, with a random variation of *M*_s_ by ±10%. The insets to Fig. 2c show the corresponding magnetization states at ±20 kA m^−1^ for a granulated structure. The pristine structure shows additional domain states that are not included in Fig. 2c.

### Data availability

All relevant data are available from the authors upon request.

## References

[1] Teufel JD (2011). Sideband cooling of micromechanical motion to the quantum ground state. Nature.

[2] Chan J (2011). Laser cooling of a nanomechanical oscillator into its quantum ground state. Nature.

[3] Kurizkia G (2015). Quantum technologies with hybrid systems. Proc. Natl. Acad. Sci. USA.

[4] O’Connell AD (2010). Quantum ground state and single-phonon control of a mechanical resonator. Nature.

[5] Camerer S (2011). Realization of an optomechanical interface between ultracold atoms and a membrane. Phys. Rev. Lett..

[6] Harris GI (2016). Laser cooling and control of excitations in superfluid helium. Nat. Phys..

[7] Forstner S (2012). Cavity optomechanical magnetometer. Phys. Rev. Lett..

[8] Wu M (2017). Nanocavity optomechanical torque magnetometry and radiofrequency susceptometry. Nat. Nanotechnol..

[9] Rath P, Khasminskaya S, Nebel C, Wild C, Pernice WHP (2013). Diamond-integrated optomechanical circuits. Nat. Commun..

[10] Hoang TM (2016). Torsional optomechanics of a levitated nonspherical nanoparticle. Phys. Rev. Lett..

[11] Mitchell M (2016). Single crystal diamond low-dissipation cavity optomechanics. Optica.

[12] Burek MJ (2016). Diamond optomechanical crystals. Optica.

[13] Li T, Yin Z-Q (2016). Quantum superposition, entanglement, and state teleportation of a microorganism on an electromechanical oscillator. Sci. Bull..

[14] Hill JT, Safavi-Naeini AH, Chan J, Painter O (2012). Coherent optical wavelength conversion via cavity-optomechanics. Nat. Commun..

[15] Losby JE (2015). Torque-mixing magnetic resonance spectroscopy. Science.

[16] Lachance-Quirion D (2017). Resolving quanta of collective spin excitations in a millimeter-sized ferromagnet. Sci. Adv..

[17] Bochmann J, Vainsencher A, Awschalom DD, Cleland AN (2013). Nanomechanical coupling between microwave and optical photons. Nat. Phys..

[18] Kim PH, Hauer BD, Doolin C, Souris F, Davis JP (2016). Approaching the standard quantum limit of mechanical torque sensing. Nat. Commun..

[19] Mohanty P (2002). Intrinsic dissipation in high-frequency micromechanical resonators. Phys. Rev. B.

[20] Hauer BD, Doolin C, Beach KSD, Davis JP (2013). A general procedure for thermomechanical calibration of nano/micro-mechanical resonators. Ann. Phys..

[21] Wohlfarth, E. P. in *Ferro*-*Magnetic Materials*: *A Handbook on the Properties of Magnetically Ordered Substances* 1st edn. Vol. 1 (North Holland, Amsterdam, 1980).

[22] Lavrik NV, Sepaniak MJ, Datskos PG (2004). Cantilever transducers as a platform for chemical and biological sensors. Rev. Sci. Instrum..

[23] Poggio M, Degen CL, Mamin HJ, Rugar D (2007). Feedback cooling of a cantilever’s fundamental mode below 5 mK. Phys. Rev. Lett..

[24] Krause, A. G., Blasius, T. D. & Painter, O. Optical read out and feedback cooling of a nanostring optomechanical cavity. Preprint at https://arxiv.org/pdf/1506.01249.pdf (2015).

[25] Kippenberg TJ, Rokhsari H, Carmon T, Scherer A, Vahala KJ (2005). Analysis of radiation-pressure induced mechanical oscillation of an optical microcavity. Phys. Rev. Lett..

[26] Wilson DJ (2015). Measurement-based control of a mechanical oscillator at its thermal decoherence rate. Nature.

[27] Yu C (2016). Optomechanical magnetometry with a macroscopic resonator. Phys. Rev. Appl..

[28] Garanin DA, Chudnovsky EM (2011). Quantum entanglement of a tunneling spin with mechanical modes of a torsional resonator. Phys. Rev. X.

[29] Qi X-L, Li R, Zang J, Zhang S-C (2009). Inducing a magnetic monopole with topological surface states. Science.

[30] Nagaosa N, Tokura Y (2013). Topological properties and dynamics of magnetic skyrmions. Nat. Nanotechnol..

[31] Kim PH (2013). Nanoscale torsional optomechanics. Appl. Phys. Lett..

[32] Michael CP, Borselli M, Johnson TJ, Chrystal C, Painter O (2007). An optical fibre-taper probe for wafer-scale microphotonic device characterization. Opt. Express.

[33] Hauer BD (2014). On-chip cavity optomechanical coupling. EPJ Tech. Instrum..

[34] Aspelmeyer M, Kippenberg TJ, Marquardt F (2014). Cavity optomechanics. Rev. Mod. Phys..

[35] Graham CD (1960). Temperature dependence of anisotropy and saturation magnetization in iron and iron-silicon alloys. J. Appl. Phys..

[36] Takahashi M (1962). Induced magnetic anisotropy of evaporated films formed in a magnetic field. J. Appl. Phys..

[37] Hughes, I. G. & Hase, T. P. A. in *Measurements and Their Uncertainties*: *A Practical Guide to Modern Error Analysis* 1st edn. (Oxford University Press, Oxford, 2010).

